# Supplementary material for: Evolution of a Cockroach Allergen into the Major Protein of Termite Royal Jelly
Source: Int J Mol Sci. 2023 Jun 18;24(12):10311. doi: 10.3390/ijms241210311 (PMC10299232; doi:10.3390/ijms241210311)
Supplement: Supplementary file 1 [file ijms-24-10311-s001.zip › Suppl. Data.pdf]

International Journal of
Molecular Sciences

Supplementary Data.

Identification and origin of the major salivary protein in higher termites

Jan A. Veenstra

Complete and partial deduced MA-1 and MA-2 transcripts from various termite species. When present signal peptides have been highlighted in yellow and the three amino acid residues methionine, cysteine and tryptophane in red.

Archotermopsidae

Zootermopsis nevadensis from genome
MKLSIIFLAVVGLACGKSLPTRSLQDDLNDFLALVPLDEVLGIALDYLAN
DEQVQDFVVYLQSEEFHKIILTVEDVKELKDFLKFINDLGIDVYEILNQV
HEILGLPPFKPDKHSRRGVGINGLINDVIAVLPTDKLKELFEEKKETSED
FKALIAAIQSPEFSNIVDALRAIKEYQDLLESLRNKGVDVDNIIALFRAL
FGLSRRGTRNLQDDLNDVLAHIPLDEVLAIGLDYLANDQEVQELVIYLQS
AEFHKIVLTVEAVPEFDEALQFVTERGIDAKAILDQIHEILGLPPRSSSK
ITRRGSGIQGLIDDLVAVIHVDHLKELFEEKLQTSQDVKDLVDFIRSSKF
EKILSTLRSIKEYQDLLQSLREKGVDVDGIIKFLKDLLGLARHY

Porotermes quadricollis worker GIAG01051081.1
FINDQGVDVYAFVNEIYEKLEKHPYQPTNTTHMGVGFYGLVNDVYSVLPL
DDWAALLDEKMETGEFFKTIVTNIRSPVLVKMVEKLQAMPEYHELLEILR
GKVVYGERIVKLLWEVSRRGTHTLRDDLKEFMALFPTDDIIQITLDYLAN
DPKLRKIYASIPYEEFPKIHAIVEHFKEYAYCLKFINDQGVDVYAIISEV
YNILDLPPYQPTNTTHMGVGFYGLVNDVYSVLPLDDW

Porotermes quadricollis worker Trinity assembled from SRR9968577
NTRRGVGVSGLINDVIAVLPIEELRALFDKKLETSEDFKALVEAIQSPEF
ATIIDSLHAIKEYQDLLEGLREKGVEVDELIALFRALFGLSRRGTRNLQD
DLNDILAHVPLDAVLSIALDYLANDAEVQELVIYLQSEEFHKIVLTVEAV
PEFRETIKFISDRGVDAEAIVNQVHEILGLPPLSKSKNTRRGVGVSGLIN
DVIAVLPIEELRALFDKKLETSEDFKALVEAIQSPEFATIIDSLHAIKEY
QDLLEGLREKGVEVDELIALFRALFGLSRRGTRNLQDDLNDILAHVPLDA
VLSIALDYLANDAEVQELVIYLQSEEFHKIVLTVEAVPEFRETIKFISDR
GVDAEAIVNQVHEILGLPPLSKSKNTRRGVG

Hodotermopsis sjöstedti (*e.g.* AB480784.1, DC235901.1, DC237539.1)
MKLPIVILAVLGLACGKSLPTRNLQDDLNDFLALVPLDQVLAITLDYLAN
DPEVQEFVVYLQSEEFHKIVLTVEDLQEFKDFVDFIEDRGIDVTGILNQI
HEILGLPPLPASKNTRRGVGVSGLINDVIAVLPIEELRALFDKKLETSED
FKALVEAIQSPEFATIIDSLHAIKEYQDLLEGLREKGVEVDELIALFRAL
FGLSRRGTRNLQDDLNDILAHVPLDAVLSIALDYLANDAEVQELVIYLQS
EEFHKIVLTVEAVPEFRETIKFISDRGVDAEAIVNQVHEILGLPPLSKSK
NTRRGVGINGLIEDLVAVIDVDLILAIFKDKQETSEDLKELLDFIRSPKF
EKILTTLRSIKEYQDLLQRLRDQGVDVDGIIKFLKDLLELNRH-

Kalotermitidae

Kalotermes flavicollis worker GHWY01015225.1
KLINSPEFRNLVEVLVSPELKELVRKLRETPESRELEEDLRDKGVDLERI
VERVKEAFRRSQRGTRSLHDDIKDILEVIPLEKIAAIAWDYVQHDEQVRE
LARHIKTPEFQERVRKVVESEEAKAIVEHLRDRGVPVDSVVSKLLGLPTS
SIRGKSTRRREGVNGLVDDVIDALPVEELKRVVKEKLINSPEFRNLVEVL

VSPELKELVRKLRETKESRELEEDLRDKGVDLERIVERVKEAFSRSQRGT
RDLNDDLNDFLALVPLDRILTILLDYLANDEEVQELILYIQSEKFHTIVL
QVEALKETKDLLQFIRERGVDVDAIANLLHEILGLPPYAVRKNTRRGVGI
NGLIDDVLAVLPIDELRALFEEKQHTSPDFKALIEAIRSPEFANILETLR
KIPEYQELLQELRDKGVEVDRIIELLKALFGLSRRGTRSLRDDIKDILEV
IPLEKIAAIAWDYVQHDEQVRELARHIKTPEFQERVRKVVESEEAKAIVE
HLRDRGVPVDSVVSKLLGLPTSSIRGKSTRRREGVNGLVDDVIDALPVEE
LKRVVK

Kalotermes flavicollis worker GHWY01103810.1
AALLDNILETKESFTTLVTVINPTVLMNMVDTVRSWPEYPEMHQIVREMV
VHVKRVSKLVWEISLRETHTIQNDVKEFMALLPTDKVLSITLDYIAKDPE
FLEAVAYIQSEEFPNIHKVVEHLKEYKECLKFINDEGVDVYAFIYEVNYI
LGLPPFPTTTTTRNGVGVYGLMKDVIAVLPLEDLKAYVDKKMENREYLKT
LVTPLKSLVLKMVKTVNTVRSWPEYKKVLEIVREKGVNEKSITEILRTFY
GPSLRGIHRLHEELKKYMALVPTDKVLEITLDYLANDQEVREFLVYIQSE
EFPPIHTIVERLKEYKDCYKFINEQGVDVYAIVNEIHDILGLPPFEPTNT
TRKGVGIIGLIDDVIAVLPLEDLAALLDKKLQTGEFFKTLVTAIHPPVFR
KMVDTVQAWPEYPEMHEILREMAVYVERMSKLLWEIYLRENHIIKNYVKE
FMTLLPTDEVLSITLDYIAKDPEFLEAVAYIQSEEF

Bifiditermes mutubae worker GHZC01020198.1
LGIDVNSIINLIHEVIGLPPYAGRKNTRRGVGINGLIDDLIHVLPIDELK
ALFEEKLKTSPDFKALIEAIQSPEFKRILDKLESLPEFQKLLQNLRDKGV
DVDRIIELLKALFGLSRRGTRNLNDDLNDFLALVPLDKVLGIVLDYLAND
AEVQELILYLQSEDFHKIVITVEELEEFKLLIKIPENLGIDVNSIINLIH
EVIGLPPYAGRKNTRRGVGINGLIDDLIHVLPIDELKALFEEKLKTSPDF
KALIEAIQSPEFKRILDKLESLPEFQKLLQNLRDKGVDVDRIIELLKALF
GLSRRGTRNLNDDLNDFLALVPLDKVLGIVLDYLANDAEVQELILYLQSE
DFHKIVITVEELE

Cryptotermes secundus from genome
MKLPIVFLAVLGLACGKSLPTRNLNDDLNDFLALVPLDEVLGIVLDYLAN
DQEVQELILYLQSEEFHKIVITVEELDEFKLLIKIPEELGIDVNSIINLI
HEIIGLPPYAARKNTRRGVGLNGLIDDLIAILPIDDLKALFEKKQQTSPD
FKALIDAIQSPEFKSILDKLESLPEFQKLLQNLRDKGVDVDHIIEFLKAL
FGLSRRGTRSLKDDLKDLLDVIPHKEIGVIVLDYVLHDREVSDLAIHVQS
PEFRQLVEAVVNSDELKDIIAHLRDQGVVVISQVNELLGFPALSLGDKAT
RRGEGVKGLVDDVIKVLPVERLKEIFRDKLLNSADFRRLIDVILSPDFKE
LVRRLKATSEFKELEDDLRNRGVDRDYIIQRLKEAFGLSQRGTRNLNDDL
NDFLALVPLDEVIAIVLDYLANDQEVQELILYLQSEEFHKILIKIPEELG
IDVNSIINLIHEIIGLPPYAARKNTRRGVGLNGLIDDLIAILPIDDLKAL
FEKKQQTSPDFKALIDAIQSPEFKSILDKLESLPEFQKLLQNLRDKGVDV
DHIIELLKALFGLSRRGTRSLKDDLKDLLDVIPHKEIGVIVLDYVLHDRE
VSDLAIHVQSPEFRQLVEAVVNSDELKDIIAHLRDQGVVVISQVNELLGF
PALSLGDKATRRGEGVKGLVDDVIKVLPVERLKEIFRDKLLNSADFRRLI
DVILSPDFKELVRRLKATSEFKELEDDLRNRGVDRDYIIQRLKEAFGLSQ
RGTRNLNDDLNDFLALVPLDEVIAIVLDYLANDQEVQELILYLQSEEFHK
IVITVEELDEFKLLIKIPEELGIDVNSIINLIHEIIGLPPYAARKNTRRG
VGLNGLIDDLIAILPIDDLKALFEKKQQTSPDFKALIDAIQSPEFKSILD
KLESLPEFQKLLQNLRDKGVDVDHIIELLKALFGLSRH

Stylotermitidae

Stylotermes halumicus worker GIAK01115845.1
PEFKNFVKFINDLGVDVYTIINTIHDVLGLPPFQPKHTTRRGVGINGLID
DVIAVLPVDDLKALFAKKLETSEDFKALVQAIQSPEFANIVETLRALPEY
QKLLQSLRDKGVE

Stylotermes halumicus worker GIAK01019216.1
QGVDVYAFISEIYYTLERHPFQPTNATRTGVGIYGLIDDVIAVLPLSDLK
ALLDKKMEPSPLDLKTLVKLIDLPAFMSFVNKVRTWPEYYEMLEIIREKD
VDVDRIIELFKELYRRGSPWLQDSLKEFMALIPTDKVLEITLDYLAYDQE
LREFIVRIQSEEFPRIHTIVEYLKKYKDCSPFISDQGVDVYEIISEIHYI
LEKHTFQPTNTTHMGVGIYGLIDDVISVLPLDDLAAFLDKKMETGEFFKN
LLTAIRSLDIMKMVDTVQKMVDTVRAWPEYQEMLEILREMVPYVEHIYKI
LRELSLRGIHRLQDDLKDFMALVQTDKVLKITLDYLHEALVYIQSEEFPK
IHKIVEYLKKYKDCSPFISDQGVYIYAIVDEVRDILGLPPVPTNITRMGV
GIYGLIDDVFAALPVEDLKTFFYMKMETREYFKVVVTAIRKTVITTIRSP
LFLKMVEIVKALPEYKKLLETLLKAGVDVVGIIKHVRVLLGLPPV

Serritermitidae

Serritermes serrifer worker GIAH01045846.1
EIALNYLSNDAEVQELVIYILSDEFLGILKTVEDLQEFKDFAKFINDLGV
DVYSIINTIHDILGLPPFQPKHIGLRGTGINGFIDDVINALPLEELKELF
ENKLVTSEDFKALVEAIQSPEFGKIVETLRSLPDYQKLLQSLRDKGVDVD
RLIELLRALFGLPNRRGTRNLQDDLNEFLALVPTDKLIEIALNYLSNDAE
VQELVIYILSDEFLGILKTVEDLQEFKDFAKFINDLGVDVYSIINTIHDI
LGLPPFQPKHIGLRGTGINGFIDDVINALPLEELKELFENKLVTSEDFKA
LVEAIQSPEFGKIVETLRSLPDYQKLLQSLRDKGVDVDRLIELLRALFGL
PPH

Rhinotermitidae

Coptotermes formosanus from genome
MKVPVVILAVLGVAFGNPLPTRSLQDDLNDFLALVPTDKVLEIALDYLSN
DKEVQEFVIYIQSEEFLKIHRTVEDLKEYKDFVRFINDLGVDVYAIINKI
HEILGLPPFEPKKDIRRGVGINGLIDDVIAVLPLEDLRALFDRKLETSED
FRALVKAIQSPEFANIVETLRALPEYQRLLQSLRDKGVDVDRIIELLRAL
FGLPPH

Coptotermes acinaciformis GHZJ01147378.1
SHLPALTSISSLKMKLPVVILAVLGVAFGNPLLTRSLQDDLNDFLALVPT
DKVLEIALDYLSNDKEVQEFVIYIQSEEFLKILRTVEDLKEFKDFVRFIN
DLGVDVYAIIDKIHEILGLPPFEPKKDTRRGVGINGLIDDVIDVLPLEDL
RALFDRKLETSEDFQALVRAIQSPEFANIVETLRALPEYQRLLQSLRDKG
VDVDRIIELLRALFGLSGRGTRSLQDDLNDFLALVPTDRVLEIALDYLSN
DKEVQEFVIYIQSEEFLKILRTVEDLKEFKDFVRFIN

Coptotermes sp. GDUG01028584.1
SEEFHKILLTVEDLQEFKDFLKFLNDLGVDVYAIINVIHDVIGLPPFQPR

HSTRRGVGINGLIEDLLAVLPLEELKALFDLKLETSEYFQALVKGIQSEE
FAKIVETLRAIPEYQDLLQKLREKGVDVDQIIALLRALFGLPREVIKAVP
F

Coptotermes sp. GDUG01030152.1
QQLIDDVLAVLPLDDLTALLDKKMETKDYFKTMVTNLKSPVFMNMVNTVR
SWPEYNEMVEILRVMVVDVEHMIQLLRELSMRETRSIQDDMKEFMALFPT
DKFIQITMDYLANDPELHEFFAYIQSEQFPKIHRAVEYLQEYGNCFKFIN
DQGVDVYAFILEIRDTFGLPPFQPTNTTRMGVGIYGFIDDVYAVLPLHDL
KAYFNMKMETRQYFKTIVTTIKTTFKIIRSPLFMNMANTVQSRPEYQKML
ETLRQRGIDVE

Schedorhinotermes intermedius worker+soldier GIAM01190288.1
FVRFISDLGVDVYAIINQIHDILGLPPFQPKHSTRRGVGINGLIDDVIAV
LPLDDLKALFENKLETSEDFRALVKAIQSPEFQKIVDELRALPEYQELLQ
GLRDKGVEVDRIIALLRALFGLSPRGTRNLQDDLNDFLALVPTDRVLEIA
LDYLSNDREVQEFVIYIQSEDFLKILRTVEGLREFKDFVRFISDLGVDVY
AIINQIHDILGLPPFQPKHSTRRGVGINGLIDDVIAVLPLDDLKALFENK
LETSEDFRALVKAIQSPEFQKIVDELRALPEYQELLQGLRDKGVEVDRII
ELLRALFGLPPH

Heterotermes malabaricus worker GHZS01013094.1
MKLPVVILAVLGLAFGSPLATRSLQDDLNDFLALVPTDRVLEIALDYLSN
DKEVQDLVVYIQSEELRDSLKTVEDLQEFKDVVRFVSEFGVDVPAIINKI
NEILGLPPFEPKQNTRRGVGINGLIDDVIAVLPVEELRALLREKLETSED
FRAFVTAIESPEFANLVNKLRESPEYQRLRESLREKGVDWDRIIELLRAL
FGQSQRGTRSLQDDLNDFLALVPTDRVLEIALDYLSNDEEVKEFVVYIQS
EEFLKILRTVEDLKEFKDFVRFISDLGVDVYAIINKIHDILGLPPFEPKQ
NTRRGVGIKGLIDDVIAVLPLEELRALFEQKLQTSPDFQALVAGIQSPEF
AKIVDTLRALPEYQDLLQKLRDKGVDVDRIIELLRALFGLPPQ

Heterotermes tenuis worker GHZU01060508.1
NTRRGVGLNGLIDDVINVLPLDKLKALFEEKLQTSEEFQALVKAIQSPEF
AKIVETLRDLPEYQNLLQSLRDKGVDVDHIIELLRALFGLSRRGTRSLQD
DLNAFLALVPTDKVLEIALDYLSNDREVQEFVVYIQSDEFLKVLATVEDL
REFKDFVRFINDLGVDVYGIIDRIHEILGLPPFEPKKNTRRGVGLNGLID
DVINVLPLDKLKALFEEKLQTSEEFQALVKAIQSPEFAKIVETLRDLPEY
QNLLQSLRDKGVDVDHIIELFRALFGLPPH

Heterotermes tenuis worker GHZU01060499.1
MKVPIIILAFLGLICGKPLPIGKLQDELNEFLVLVPTDKILEIALDYLAY
DPEVKEFVVYIQSEEFPKIHRVVEYLKEYKDCFKFFNDQGVDVYAIVNEI
HDILEEHPFQPTNTTRTGVGFYGLIKDVVAVLPLDDWTALLDKKLEAGDL
FKTLVTATPVFMKMVDNVRTWPEYHELLEILREMVVKVEPSIELLWEIFL
RETRSLQEDLHDFMALIPSNKIFDIVLDYLLNYKELQEFFVYIRSEEFPK
IHKIVEYLKENKCFKFYNDEGVDFYVIINEIRDILDLPPFQPTNTTRTGV
GIYGLVDDVIGVLPLDDMKALFDKKTETREYFKTLVKVLKSPVLVKMVHT
VRYSFLPKMVDTVRSLPEYHEVLEILREKVVDVDRIVELFRKLYWRGTHK
LQYELKELMALFPTKKVLEIALDYLAYDEEVRQFLVYIQSEKFPKIHKIV
EHLKEYKDCLKFINDEGVDVYEIINEIYDNLGFPPFEPTNTTRTGVGIYG
LIDDVIAVLPLDLTALLGKKIETKEFVKALVSVIHSPVFVNMVNTVRAWP
EYNELLNILRVTVVDVKPIIKLLWELSLRGTHVIQDDLKEFMALVPTDKI

IQITLDYLANDPELHKMFVYLQSEEFPKIHKIVEYLKKYKYCLTFIDEYG
VDVYAFVLEVRDILGLPPFEPTNTTHMGVGIYGLIDDVFAVLPVDDLKAL
YHMKMKTREYFKVVVTTIRSPLYVKMVETVKALPEYKKLVEILREMGVDV
VGIIKHVRVLLGLPPI-

Prorhinotermes inopinatus GHYS01118366.1
NIVDTLRALPEYQRLLEGLREKGVEVDRIIELFRALFGLSRRGTRSLQDD
LNDFLALVPTDKVLEIALDYLSNDQEVQEFVVYIQSEEFLKILKTVEDLK
EFKDFVRFINDLGVDVYAIINQIHDILGLPPFQPKKSTRRGVGIDGLIDD
VIRVLPLDDLRALFDKKLETSEDFQALVKAIQSPEFANIVDTLRALPEYQ
RLLEGLREKGVEVDRIIELFRALFGLPPH

Reticulitermes speratus from genome
MKLPIVILAFLGLALGKSLPTRSLQDDLNDFLELVPTDKVLEIALDYLSN
DSEVQEAVVYIQSEEFLTILRTVEELRNLKTFVRFLSDHGIDVVAIINRI
HDILGLPPFQPKQSTRRGVGINGLIDDVIAVLPLDDLRALFDEKLENSED
FKALVEAIQSPEFANIVNTLRALPEYQRLLENLREKGVDVDRIIELLRAL
FGLPPRRGTRSLQDDLNDFLALVPTDKVLEIALDYLSNDSEVQAAVVYIQ
SEEFLTILRTVEELQEFKDFVRFLSDHGIDAVAIINRINDILGLPPFQPK
QSTRRGVGINGLIDDVIAVLPLDDLKALFNEKLENSEDFRALVEAIQSPE
FANIVNTLRALPEYQRLLENLREKGVDVDRIIELLRALFGLPPRRGTRSL
QDDLNDFLALVPTDKVLEIALDYLSNDSEVQAAVVYIQSEEFLTILRTVE
ELREFKDFVRFLSDHGIDVVAIINRIHDILGLPPFQPKQSTRRGVGINGL
IDDVIAVLPLDDLRALFDEKLKNSEDFRALVEAIQSPEFANIVNTLRALP
EYQRLLENLREKGVDVDRIIELLRALFGLPPH

Reticulitermes aculabialis GHMS01001114.1
FLALVPTDKVLEIALDYLSNDSEVQEAVVYIQSEEFLTILRTVEELREFK
DFVRFLRDHGIDAVAIINTINDILGLPPFQPKHSTRRGVGISGLIDDVIA
VLPLDDLKALFEKKLETSEDFRALVEAIQSPEFANIVDTLRALPEYQTLL
EKLREKGVDVDRIIELLRALFGLPPRRGTRSLQDDLNDFLALVPTDKVLE
IALDYLSNDSEVQEAVVYIQSEEFLTILRTVEELREFKDFVRFLRDHGID
AVAIINTINDILGLPPFQPKHSTRRGVGISGLIDDVIAVLPLDDLKALFE
KKLETSEDFRALVEAIQSPEFANIVDTLRALPEYQTLLEKLREKGVDVDR
IIELLRALF

Termitogeton planus nymph GHXB01007738.1
YGLINSVNDILGLPPLKPMKSTRRGVGLDGLITDVIAILPVADLEELFAK
KLATSPDFQALVEGIQSPEFARIVEVLRGLPEYQDLLNNLRAKGVDIDHF
IELLRALFGLSRRGTRNLQDDLNDFLALVPVDRVLEIALDYLSNDKEVQE
FVIYIQSEEFVKILRTLEDLQEFKDFIKFLSDLGVDAYG

Termitogeton planus nymph GHXB01011760.1
MKIQIVIWAVLGLACGKYLPTVPLEDDLKEFMTLVPKDKLFEIALEYLYH
DREVKEFVVYLQSEEFPKIHTFIEYLKEYKEVKVPCLKFFDEYGFDVYEI
VHHIHDVLHLPPFEPTNRTYMGVGIHGLIEDVISVLPLKELKVLYEKKLE
TREYFKTLVTTIKSPVFKSVLDKVRTWPEYYKLLEILREKFVDLDHIHEL
LYELYWRGIHILEHELKEFIPLDPTAKVLDILLDYLTYDPELKEFIVYIQ
SEEFPKIHKIVEYLKKYKELYVPCLKYIDEYGVDVYAIVHEIYDIISEIY
IILGLPPLEPTYTTRMGVGIPGLIEDVITVLPLDELKVLIEKKVETKEYV
KILYTVIHSPEFMEMVYTLRVLPEYKELVEFLLKIGVDVGRIIELVRTLL
SLPRL

Termitidae

Jugositermes tuberculatus worker GHZX01073273.1
MKLPIVILAVLGLAYGKSIPTRNLQDDLDDFLALIPLDKVLEITLDYLAN
DPEVQEAVIYIQSEEFHTIVTTVEALPEFKTLVKFIGDLGIDIYTIINTI
HDIIGLPPYQPKHSTRRGVGINGLLDDLIAVLPVEELKALYEEKLKTSPD
FQALIAAIQSAEFATILETLRALPEYQKLLDSLREKGVDVDRIIEFLKAL
FGLSRRGTRNLQDDLDDFLALVPLAQVLEVALDYLANDAEVQEFMVYLQS
EEFYKIIDAVEKLREFRNFLELINGLGVDIYTIINTIHDILGLPPFVPKY
NTRRGVGIKGLLDDLIAVIPLDDLKALFEQKKQTSPDFQVVIAAIQSPEF
KVIVDTLHALPEYNALLDNLRAKGVDVDHIIAVLRELFGLPP

Jugositermes tuberculatus worker GHZX01073285.1
MKLPIVILAVLGLACGKPLPTGKVQDDLKEFLELVPTDKVLEIALDYLYY
DKEVKELVVYIQSEEFPPIHKIVEYLMEYKDCLKFINEYGVDVYAVIKEI
HDILESHPFEPTNTIRMGVGIYGLIDDVIAVLPLDELKALFEKKMETREY
FRTLVTPFKSPVFMKMVDTVRSWPEYQELLEILRGKVVDVDRTIELLRVL
YWRGTRKIQEDVKEFLKFVPQHKVFEIVLDYLSNDKEVKEFVVYIQSEEF
PYIHKIVEYLKEHKACFKFIDEYGVDVYAIIKEIHDILRFPPFEPMNTTR
MGVGFQGLIDDVIAVLPPLDGLKALYEKEMEIRECLKTLYTAIYSPVFRK
MVDILRDWPEYQEILDYLQGKEQEILDYLRGKGVDVDPYIELLRTLYSLS
WRGIHILQEDMTEFMALVPTHKFFEIALDYLAYDPEVQKFIVYIQSEEFP
MIHKMVEYLKEYKDLLKFIDEHRVDVYAIIKKIHDTLGFPPFEPTNTTRM
GVGIHGFIDDVIAVFPLDDLKTLVVKKMETREYLRPLVPVYKTVVKTVRS
PEFKKMVDTLRAMVDTVQTWPEYQKLVQMLRDKVFDEDHIIELFTELREL
FWRGTHILKDDLTEFMTLVPTDKVLDITLEYLAYDPKLKEFFVYIQSEEI
PMIHTIVEYLKEYKDLLKFIDEHRVDVYAIIKKIHDTLGFPPFEPTNTTR
MGVG

Leptomyxotermes doriae worker GHZY01097234.1
MKFPIVFLAVLGLVCGKSLPTRNLQDDLNDFLALVPLDKVLEISLDYLAN
DQEVQDAVIYLQSPEFHKILTTVEDLKEFKDFLKFVNDLGVDVYSIINTI
HDVLGLPPFQPKHSVRKGVGLNGLIDDVIDILPVDELKALFDKKLQTSPE
FQALVAGIQSPEFKNILDNLRAVPEYQDLGQSLQEKGVDVDHIIELLKAL
FGLSRKGTRTLQDDLNDFLALVPLDKVIEIALDYLANDQEVQEAVIYLQS
PEFHKILTTVEDLKEFKDLVKFINDLGVDIYSIINTIHDVLGLPPFQPKH
SVRKGVGLNGLIDDLIDVLPVDELKALFDKKLQTSPEFQALVAAIQSPEF
KNIVDTLRAIPEYQDLLQSLRDKGVDVDHIIELLKALFGLSRKGKSLPTG
TLQDDLNDFLALVPIDKVLEISLDYLANDQEVQEAVAYLQSEEFYMILTT
VEDLKEFKDFVEFMNDLGVDVYALDN

Cavitermes tuberosus worker GHZE01030677.1
MRELFHLLALMKLPIVFLAVLGLVWGKSLPTRTLQDDLDDFLALIPLDKV
LEIALDYLANDPEVQDAVIYLQSEEFHKILTTVEALQEFKNFVKFINDLG
VDVYTIINTIHDILGLPPFQPKHSTRRGTGLSGLINDILDILPLDDLKDL
FNKKLETSPDFKALVAGIQSEEFAKIVDTLRALPEYQVLLQNLRDKGIDV
DQIIQLLRSLFGLPQKGSRTLQDDLDDFLALIPLDKVLEIALDYLANDPE
VQEAVIYLQSEEFHKILTTVEALQEFKNFVKFINDLGVDVYTIINTIHDI
LGLPPFQPKHSTRRGTGLSGLINDILDILPLDDLKDLFNKKLETSPDFKA
LVAGIQSEEFAKIVDTLRALPEYQVLLQNLRDKGIDVDQIIQLLRSLFGL
P

Cavitermes tuberosus GJZF01090478.1
VDVYAFILEIHDFLHLPPLEPTNTPPMGVGFLGLVRDVIAVFPLDDLKVV
FDKTEIRKYFKTLVTAVHSLFVNVVDTVKTWPEYEKLLEIIREKVFDVDR
IIELFRELYLRGNHKLQEDLKEFMALLPSNKVFEIVLDYVSYDEKLREFI
VYIQSEKFPPIHKIVEHLTEYKDCLKFINDEGVDVYAIILEIHDILGLPP
IEPTNTIPMGVGIYGLIRDVVAVLPLDDLTVFLDKKLGTKDFFMTLITAI
RSPVLMDMVNIVRSWPEYHELLKILRGMVVDVEPIIKLLKETLLRETHSI
RDDVKEFMILFPTDKVIHITLDYLANDTRLHEIFAYIQSEEYPIIPKIVE
YLKEYKYCLKFITDEGVDVYAIILEIHDILGLPPIEPTNTIPMGVGIYGL
IRDVVAVLPLDDLTVFLDKKLGTKDFFMTLITAIRSPVLMEMVNIVRSWP
EYHELLKILRVMVVDVKPIINLLKETLLRETHSIRDDVKEFMILFPTDKV
IHITLDYLANDTRLHEIFAYIQSEEYPIIHKIVEYLKEYKHCLKFIDDQG
VDVYAFILEIHHILGLPPLEPVDTTRTGVGIFGLIDDVYAVLPLEDLKEF
FHLKMKTREYFKILVTSLKTTVTTIRSPVIVNMVDTVRALPEYQKLIEIL
LEMGVDVVLIIERLKVLYALLPF

Inquilinitermes inquilinus GKIC01204865.1
MKLPIVFLAVLGLVCGKSLPTRNLQDDLNDFLALVPLDKVLEIALDYLAN
DPEVQEAVIYLQSEEFHKILTTVEALPEFKNFVKFISDLGVDVYTIINTI
HDILGLPPFQPRHSTRRGVGLSGLIQDITAVLPLDDLKALFENKLQTSPD
FQALVAGIQSEEFSKIVNTLRALPEYQELLQTLRDKGVDVDQIIALLRAL
FGLPQKGTRSLQDDLDDFLALIPLDKVLEIALDYLANDPEVQEAVIYLQS
EEFHKILTTVEALPEFKNFVKFISDLGVDVYTIINTIHDILGLPPFQPRH
STRRGVGLSGLIQDITAVLPLDDLKALFENKLQTSPDFQALVAGIQSEEF
SKIVNTLRALPEYQELLQ

Inquilinitermes inquilinus GKIC01076654.1
NMVDTVRAWPEYKKLFDSLRRRGVDVERIIEHLRALFGLSWRGNNWLQDD
LKEFLALVPTDKVLEIALDYLANDQEVREFVEYIQSEEFPKIHTIVEHLK
QYKDCLKFINDQGVDVYAIINEIYNFLDLPPFQQTNTTRTGVGMQGLVDD
VIAVLPLDDLKALFGKTETRKYFKAMFTAIHSPAFMNMVDTVRTWPEYNK
LQQMLREKVFDVDRIMELFRELYWRGTHRLQDELNEFMEHLPTDKFLEIL
LDYLANDEEVREFAVYIQSEEFPKIHKVVEYLKEYKYCLRFINDEGVDVY
ELILEIHDFLNLPPLEPTTTTRMGVGFLGLVHDVIAVLPFDDLKAFFDKT
EITKYFKTLVTAIQPHYVNVVDTVRTWPEYKKLLELIREKVVDVDRYIEI
FRELYWRGINKIQDQQYLQYVQYIQDYLKEFMAIFPTNKVFEIVLDYLAY
DKQVREFLVYIQSEEFPLIHKIVEYLKEYKYCLKFITDNGVDVYTFIL

Embiratermes neotenicus worker GHZN01085478.1
IALDYLANDPEVQEAVIYLQSEEFHKILTTVEALQEFKNFVKFINDLGVD
VYTIINTIHDIIGLPPFQPRHSTRRGVGLNGLFDDLTAVLPLDELKALFE
EKLQTSPDFQALVAGIQSPEFAKIVETLRALPEYQNLLQSLRDKGLDVDR
LIELLRALFGLSRRGTRTLQDDLNDFVALIPVDKVLEIALDYLANDQEVQ
EFVTYLQSDEFHVIINTIEGLQEFRDFLKFLNDLGVDVYAIINTIHDIIG
LPPFQPRHSTRRGVGLNGLFDDLTAVLPLDELKALFEEKLQTSPDFQALV
AGIQSPEFAKIVETLRALPEYQNLLQSLRDKGLDVDRLIELLRALFGLPT
F

Euhamitermes sp. worker GHZO01132964.1
MKIPIVILAVLGLACGKSIPETRTLQDDLNDFLALVPTDEVLAIALDYLA
NDVEVQEAMIYLQSEKFHTIVLTIEGLQETKNFLEFIDNTGLDIYLYLNT

LHDFIGLPPYVPKHSIRRGVGINGLLQDIIAILPVEDLKALFDEKLQTSP
DFQAFIASIQSEEFAVILDTLRALPQYQELLNSLSEKGVDVAAIIEFLKT
LFGLSRQGTRTLQDDLDDFVALLPVDVLLGIALDYLANDAEVQEFMVYLQ
SPPFHLIITTEEALPEFKNFVDFIVVHGVDAYGIINTVHDILGLPPYGSK
YHTRRGVGINGLLQDVIANLPLDELKALFDDKKGTSPDFQDLLAAIQGPE
FTVIVNTLHALPEYNSLLDDLRAKGVDVDGIIAALRELFGLPPL

Euhamitermes sp. worker GHZO01109466.1
VKEFVAYMHSEKFPYIHKPVEYLKQYKLCLKHIDVYAIINKVYDTLGLPP
FEPTNNARTGVGFLGLKDDMIAVFPLDGLKALVEKKMETREYYKPLLPAY
KTLVKIIKSHEFKKIVDTVRARMVDTVRPMMVDLVRNWPKYWPEYQKQFE
MLREKYFNKDHIIEKLGKFRELFLKGIHYLKEDITEFMELVPPHKFFEIS
KEYLTYDPEVQEFVAYIQSEKFPFIHKPVEYLKQYKPCLKLIDVYAIINK
IYDTLGLPPFVPTNTTRTGVGFHGLKDDMIAVFPLEDLNAFFEKKMETRE
YYKPLLPAYKTLVKTIQPHVLKKIVNTVRLIMVDLVQPMMVDLVQPMMVD
LVQNWPKYLSEYQKQFVMLREKYFNKDRIVHKLGELRDLFLQGKYILKED
LQELNSLVPTKKVLIITLDYVAYDPELQELLDYIQSEKFPIIHNFVGYLK
GYKDRLQFIDDRGVNIYAIVKIIYDTLGYPLPEPTNTTHSGIQGLMPKVI
ASFPLEDLNAFIEKKMEAREYYKPLLPVYKTLVKTIESPVLKKVVVTVRA
RMVDTVRPMMVDLVRNWPQYWPEFQKQFEMLRENVFDKDRVIELLRELRE
LFLQGAYILKDDLQEFMPLVPAGKVCDIILVYLAYDPEVQEFLDYIQSEE
FPVIHNIVEYLKGYKDHLQFIDDHGVNIHAIIKIFYDTLGYPPLEPTNTT
PNGIQGLKQNVIAAFPLEELKAFVAKKIETRPYFRTMVSTYNTLVTTIQS
PEFRNMVYAVRSFMVDTVGSWPEYQEVVEMLREKAFDVNRTIELLRDLSK
LFWRGAYILIDDLQEFMSLVPTDKVLDITMDYLTYDPEVQEFLDYIQSEE
FPMIHNIVEYLKEYKDRLPFINDHGVDFYAIIKQVHDTLGYPLFEPTNTT
RTGVGFHGLIHDVIAAFPLDELKAFVENKLETRNYFKILVTVYKTLVATL
QSPEFREMVDTVRAFMLDTLQAFTEHQRLLEILHEMGVDVDRIIELLRKL
FNWTPF

Anoplotermes banksi worker GHYX01127898.1
MKIQIVILAVLGLACGKSIPTRTLQDDLNDLLALIPLDELLGIALDYLAN
DEQVQETVIYLQADEFHKIVLTIEGLQETKNLLQFVNDLGVDVYSILNTL
HDIIDLPPYQPSRSTRKGVGLNGLIDDIFAILPTDALQALYNDKLQNSPD
FQALVAALQGPEFATILETLRALPEYQGFLQFLSDKDVDVDRIIALFKAL
LGISRQGTGTRTLQEDMDDFVALVPLDAVLTVALDYLANDQEVQDFMVYL
QSDPFYKIIGTVEALQQFKDFLVIINDLGIDVYSIINTIHDILGLPPYVP
KHNTRLGVGISGLLNDELALLPLDDLKALFEQKKQTSADFQAVVSAIQSP
NFAVIVDTLHGVPEYNALLNSLRDKGVDVDSIIAALRHLFDLPTF

Microcerotermes sp. worker GHYV01038129.1
MKFPIVFLAVLGLVCGKSLPTRSLQDDLNDFLALVPLDKVLEIALDYLAN
DPEVQEAIIYLQSDEFHKIITTVEALPEFRDFLEFLNGLGIDVYAIINTI
HDVLGLPPFQSRHSTRRGTGVNGLLEAVIDVLPLDELKALFDKKLETSED
FKALVKGIQSPEFAKIVDTLRGIPEYQDLLKSLKEKGVDVDRIIELLRAL
FGLSRRGKSLPTRSLQDDLNDFLALVPLDKVLEIALDYLANDPEVQEAII
YLQSDEFHKIITTVEALPEFRDFLEFLNGLGIDVYAIINTIHDVLGLPPF
QSRHSTRRGTGVNGLLEAVIDVLPLDELKALFDKKLETSEDFKALVKGIQ
SPEFAKIVDTLR

Pseudacanthotermes militaris worker GHYT01045980.1
EYQNLLDSLRAKGVDVDRIIEFVRTLFGVSRRGTRSLQDDLDDFLALLPR

DQIVSIALDYLANDQEVQDLVVYAQSEEFHNIVTTVEALPEYKNFVKFLS
DHGLDVTSFIDTIHDAIGLPPYQPRHSTRRGVGISGLINDLIALIPVDKV
KALFEEKLETSPDFKAFVEAIQSPEFQSIVETLRALPEYQNLLDSLRAKG
VDVDRIIEFVRTLFGVSRRGTRSLQDDLDDFVALLPLDQVIDIVLDYLAN
DQEVQEFVVYIQSDEFHKIVTTVEALAEFKNFVKFLSDHGLDVTSAINTI
HDAIGLPPYQPRHSTRRGVGISGL

Occasitermes sp. worker+soldier GIAP01117651.1
FVKFISDLGIDVYTIINTIHDILGLPPFQPKHSVRRGVGLNGLIDDVINV
LPLDDLKALFEQKLQTSPDFKALVAGIQSPEFANILNTLRAIPEYQDLLQ
TLRDKGVDVDHIIELLKALFGLSRKGTRTLQDDLNDFLALVPLDKVLEIA
LDYLANDPQVQDAVIYLQSPEFHKILTTVENLKEFKDFVKFISDLGIDVY
TIINTIHDILGLPPFQPKHSVRRGVGLNGLIDDVINVLPLDELKALFEQK
LQTSPDFQALVAAIQSPEFANIVATLRALPEYQDLLQTLRDKGVDVDRII
ELLRALFGLSQ

Occasitermes sp. worker+soldier GIAP01117649.1
FITDQGVDVYEIISEIHYILEKHPFQPTNTTRMGVGFYGLIDDVIAVLPL
DELAAFLDKKLITGEFFKNLLTAIRSLDVMKMVDTVRAWPEYREMLEILR
EMVPYVERIYKLLWEPSLREIHRLQDDLKKFTALFPTDKVLEIIRDYLES
DPELHKVLVYIQSEEFPKIHTIVEDLKEYKDCFKFITDQGVDVYEIISEI
YYILEKQPFQPTNTTRMGVGIYGLIDDVMAVLPLDDLTALLDKKLETREL
FRSLLTAIRSLDVMKMVDTVRAWPEYHEMLEILQEMVPYVERIYKLLWEP
SLREIHRLQDDLKKFTALFPTDKVLEIIRDYLASDPELHEVLVYIQSQEF
PRIHKIVEYLKEHKDCFKFISDQGVDVYAIISEIHYILERHPYQPTNTTR
TGVGIYGLIDDVIAVLPLSEWKALLDKKMEASQYLKTLVKLIDIPAFMSI
VDTVRSWPEYYELLEIVREKDVDVHRIIGLFKELYKRGTPWLKDSLKELM
AIIPTDMVLEIALDYLA

Sphaerotermes sphaerothorax worker GIAI01046998.1
IHDILGLPPFQPKHSTRRGVGINGLIDDVLAVLPLDELKALFDQKLQTSP
DFQALVKAIQSPEFAKIVDTLRAMPEYQDLLQKLRDKGVDVDRIIELLRA
LFGLSRRGTRTLQDDLNDFLALVPTDKILEIALDYLANDPEVQEAIIYIQ
SEEFLKILTTVEALQEFKDFVKFINDLGVDVYAIINAIHDILGLPPFQPK
HSTRRGVGINGLIDDLIAVLPIDELKALFDQKLQTSPDFQALVKAIQSPE
FAKIVDTLRAMPEYQDLLQKLRDKGVDVDRIIELLRALFGLSRRGTRTLQ
DDLNDFLALVPTDKILEIALDYLANDPEVQEAIIYIQSEEFLKILTTVEA

Astratotermes sp. worker GHYY01048487.1
MKIPIVILAVLGLVCGKSIPTRTLQDDLDDFLALIPVDKILEIALDYLAN
DPEVQDAVIYLQSDEFHKIVLTVEGLQETKDLLKFVYDLGLDVYAILNTI
HDIILLPPFVPKHSTHRGVGINGLLDDILAVLPLDELKALYDEKLQTSPD
FQALIAAIQSPEFATILDTLRALPEYQKLLETLREKGVDVDRIIELLKAF
FGLSRRGTRTLQDDLDDFLALIPVDQILGIALDYLANDPEVQDAVIYLQS
QEFHSIVLTVEGLQETKDLLKFVYDLGIDVYAILNTIHDIILLPP

Spinitermes trispinosus worker GIAJ01182109.1
MKLPIVFLAVLGLVWGKSLPTRTLQDDLDDFLALVPVDKVLEIALDYLAN
DPEVQEAVIYLQSEEFHKILTTVEALQEFKDFVKFLNDLGVDVYTIINTI
HDILGLPPFQPRHSTRRGTGLSGLIDDILAVLPLDDLKALFNEKLQTSPD
FQALVAGIQSEEFSVIVNTLRAIPEYQDLLQKLRDKGIDVDKIIELLRSL
FGLPHKGTRTLQEDLDDFLALIPLDKVLEIALDYLANDPEVQEAVIYLQS

EEFHKILTTVEALQEFKD

Spinitermes trispinosus worker GIAJ01133998.1
MKLPIVFLAVLGLVCGKPVPTRSLKDDLKEFLALVPTDKVLEIALDYLAY
DPEVKEFVVYIQSEEFPPVHKIVEHLKEYKDCLRFINDEGVDVYAFINEI
HDFLGFPPYEPTNTTPMGVGIYGLIRDVVAVLPVDDWAVLLDKMLETKES
FQTLVTVINPTVLLNMVDTVRSWPEYHELLKILQEKFVEVHHIFELLKEL
YWRESRSLLDDLHDYMELVPKNKVFEIVLDYVANDPEVKKFIVYIQSEVL
PMIPKIVEYLKEYKHCLKFINDEGVDVYAFILEIHDFLHLPPLEPTNTTR
MGVGFLGLIRDVIAVLPFDDFKAVFDKTETRKYFKTLVTAIHSLFVNVVD
TVRMWPEYEKLLEIIREKVVDVDRIIELFRELYLRGNHKIQDDLKEFMAL
VPTNKVFEIVLDYLAYDEEVREFVVYIQSEEFPPIHKIVENLKEYEYCLK
FINDEGVDIYAFILEIHDFLHLPPLEPTNTKRMGVGFLGLVHDVIAVLPL
DDLKAVFDKTETRKYFKTLVTAIHSLFVNVVDTVRTWPEYEKLLEIIREK
VFDVDRIIEILRELYLRGNHKLQEDLKEFMALVPTNKVFEIVLDYVAYDE
ELREFILYIQSEKFPPIHKIVENLKEYEYCLKFINDEGVDVYAFILEIHD
ILGLPPFEPIDTTRMGVGIYGLIDDVYAVLPLEDLKAFIDMKIKTREYFK
ILVTSLKTTVTTIRSPVILNMVDTVRALPEYQRVLVILRALPEYQRVLEI
LREMGVDVVLIIERLRVLYGLLPY

Palmitermes impostor worker GIAD01002139.1
FPPIHKIVENLKEYKYCLKFINDEGVDVYAIILEIHDILGLPPFQPRHST
RRGTGLNGLINDILDILPLDDLKDLFNKKLETSPDFQALVAGIQSEEFAK
IVDTLRALPEYQDLLQKLRDKGIDVDQIILLLRSLFGLPRKGTRTLQDDL
DEFLALIPLDKVLEIALDYLANDPEVQDAVIYLQSGEFHKILTSVEDLQE
FKDFVKFISDLGVDVYTIINTIHDILGLPPFQPRHSTRRGTGLNGLINDI
LDILPLDDLKDLFNKKLETSPDFQALVAGIQSEEFAKIVDTLRALPEYQD
LLQKLRDKGIDVDQII

Palmitermes impostor worker GIAD01002133.1
MKVPIIILAFLGLVCGKPLPSGSLQDELNDFLVLVPTDKIFEIALDYLAN
DPEVKELVVYIQSEEFPKIHRVVEYLNKYKDCLKFISDNGVDIYAIINEI
HDTLERHPFQPTNTTHKVVGIYGLIDDVAAVLPFNDLMTLFDKKMETSKN
LKTLVTIIHSPVFMDMVNAVRAWPEYHKLLEILQEKVVEVAHIIELLREI
SWQGTNSLQDDLKDFVAIFPTDRVLEIALDYLSSDQEVKKFVVSIQSEEF
PKIHKVFEYVKEYKDCLKFINDQVVDVYAIIKGIHDILERHPFQPTNTTR
MSVGIYGLIDDVVAVLPINELKTLFDKKMKTSKDFRTMVTAIHSPDFANM
VETVQAWPEYKKLFESLRRKGVDVERITEHLSALFGLSMRGTHRLQDDLK
EFMALVPTDKVLEIALDYLANDQEVREFVVYIQSEEFPKIHKIVEHLKEY
KDCLKFINNQGVDIYALINEIHDIPERHPFQPTNTTHMGVGIYGLIRDV

Cephalotermes rectangularis worker GHZD01100093.1
KIVETLRAIPEYQELLQNLREKGVDVDLIIEFLRALFGLSRKGTRTLQDD
LNDFLALVPLDKVLEIVLDYLANDAEVQEAVIYLQSAEFAKILTTVENLQ
EFRDFVKFINGLGVDVYAIINAIHDVLGLPPFQPRHSTRRGVGINGLLQD
LIAVLPVDELKALFEQKLQTSPDFQALVAGIQSAEFAKIVETLRAIPEYQ
ELLQNLREKGVDVDLIIEFLRALFGLSRKGTRTLQDDLNDFLALVPLDKV
LEIVLDYLANDAEVQEAVIYLQSAEFAKILTTVENLQEFRDFVKFING

Macrotermes natalensis MA-1 from genome
MKLPIVILAVFGLVCGKSLPTRDLQDDLNDFLALLPTDQIVEVALDYLAN
DQEVQDFVVYIQSDEFHGIVTTVEALDEFKNFVKFIDGLGVNLTQIINTI

HDVIGLPPFQPRHSTRRGVGVSGLVDDVLALIPIDKIQALFEEKLQTSPD
FQAFVQAVQSPEFQGILNTLRALPEYQNLLDNLRAKGVDVDAIIEFLRSI
FGVSRRGNRNLQDDLNDFLALLPTDQIVEVALDYLANDQEVQDFVVYIQS
DEFHGIVTTVEALDEFKNFVQFIDGLGVNLTQIINTIHDVIGLPPFQPRH
STRRGVGVSGLVDDVLALIPIDKIQALFEEKLQTSPDFQAFVQAVQSPEF
QGILTTLRALPEYQNLLDNLRAKGVDVDAIIEFLRSIFGVGSL

Macrotermes natalensis MA-2 from genome
MRISIVFLAVLGLVCGKPVENGSLQDDLNDFMALVPKDKVLEITLDYLHN
DKQVQEIVAYVQSEEFPLIHTVVEYLQEYKDCLKFVNDHGVDVYAIVNEI
HDRLGYPPFQPTNNTRTGFGINGLFDDVIAVLPRDDIIALVYQKKETIQL
YRALYTVIRTPEYTNLMDTVRTLPEWKELRNILQQRGVDVERYFERIKEF
FGLSRRGTRSLHEDVNDLIALVPTDKVLDILLDYLYNDEKVQKFVAYMQS
EEFPLIHTVVEYLQQYKNCLKFVNDHGVDVYAIVSEIHDRLGYPPFQPTS
FQPTNNTRTGVGINGLFDDVIAVLPRDDIIALVYQKKETIQLYKALYTVI
RTLEFTNLVDTVRTLPEWKELRSILLQEGLDVDYYIERIKEFFGLSRLGI
SRLQDDLNDLMALVPKDTVLEIVLDYLYNDEEVQEFIAYVQSEEFPLIHT
LVEYLQQYKNCLKFVNDHGVDVYAIVSEIHDRLGYPPFQPTNNTRTGFGI
NGLFDDVIAVLPRDDIIALVYQKKETIQLYRALYTVIRTPEYTKFVNTVR
TLPEWKELRNILQQRGVDVERYIESIKEFFGLSRRGTRSLHEDVNDLIAL
VPTDKVLDILLDYLYNDEKVQKFVAYVQSEEFPKIHTLVEYLKQYKNCVK
YIDDSVIDIYAIVNEIHDSLGYPPFQPTNNTRTGVSINGPIVDMIAAVLR
KDINFAVLPRDDMNALVYQKKETSQLYKTLYTVIRTPEYTNLVDTVRTLP
EWKELRNILQEWGVDVDHIYELFRALFVQP

Aparatermes sp. worker GHYZ01043183.1
ILNTLHDVIGLPPYQPSRSTRRGVGLNGLIDDIIAILPVDKLQALFIEKL
QTSVDFQALIANIQGPEFAAILDELRALPEYQQFLQDLREKGVDVDHIIE
LLKALFGLSRRGTRNLQNDLDDLLALVPLTDVLAVALDYLANDLEVQDFM
LYLQSAPFYKIIGTVEGLQQFKDLLVIINNLGVDPYAIVNTIHDILGLPP
YVPKHSTRKGVGINGLLTDVLALLPLDDLKAIFEQKKQTSVDFKALVSAI
QSRDFAVIVDTLHGLSEYNSLLNNLRAYGVDVDGIIAYLRQLFDLPEF

Aparatermes sp. worker GHYZ01043168.1
MKFPVAFLAVLGLVCGKPLPIAKFQEELNDFLVLVPTDKILEITLDYVAN
DTEVKEFIAYIQSEEFPKIHTILEHLQEYKECFPFITDQGVDVYELISEI
YYVLEKHPFQPTNTTRMGVGFYGLITDVMAVIPLDDLTALLDEKLETGEL
FRSLLTEIHSLDVMKMVDTVRTWPEYQEILEILREMIPYVERIYKLLLEP
SLRGIHRLQDDLKNLTALFPTEKVLEITLDYLANDPKLHELFVYLQSEAF
PRIHKVVEYLKEYKNCFKFINEEGIDVYAVLSEIHYILEGHPFQPTNTTR
TGFGIYGLIDDVIAVLPLSVWKTLIDKEMEASPYLKTLVKLLDFPAFMSI
VNKVRSWPEYYELLEIIREKDVDVDRITELLKELYRRGNPWLRESLKELM
TLIPTDKVLEIVLDYLAYDQEVRGFIVRIQSEEFPRIHKTVGYLKEYTYC
FKYINEQGIDIYQIISEIHYILEKHPFQPTNTTRMGVGFYGLIDDVIAVL
PFDDLAAFLDTKMETGEFFKNLLTAIRTLDIMKMVDTVKKIVDTVRTWPE
YQEMLEILREMVPYVENIYNILKELSLRGIHSLQDDLKELMVLVPTDKVL
EITLDYLHEALVYIQSEEFPLIHKIVVYLKKYLDCFPFITDEGVDIYVIV
NIVRDILGLTPIPPTNITHMGLIHDVIAVLPLDDLKVLLDVKMETRVYIR
TLVKIIRSPVLVNMVETVKKMVETVRALPLYQRLLEILREKGVDVELIIE
HLRVIFYPPRP

Basidentitermes aurivillii worker GHZB01184459.1

ILSTVEGLQEFKDVVKFISDLGVDVNAIINQIHDFLGFPPFQPRHSTRRG
TGINGLIDDLLAILPLEDLKALFDSKLETSEEFNALITGILSEEFAQIVE
TLRARPEYQDLIQKLVEKGVDVDRIIELVKALFGLSRKGTRTLQEDLDDF
LALIPVDKVLEIALDYLANDLEVQEAVIYLQSEEFYLILSTVEGLQEFKD
VVKFISDLGVDVNAIINQIHDFLGFPPFQPRHSTRRGTGINGLIDDLLAI
LPLEDLKALFDSKLETSEEFNALITGILSEEFAQIVETLR

Basidentitermes aurivillii worker GHZB01184433.1
YKNCLKYVNDRGVDVYAVIKKVYDTLPFPPFEPTNTIRTGVGFHGLKNDM
IAAFPLSELKALVEKKMETRKYLKPLLPAYKIMVKTIQSPEFKEMVENVQ
ETVRTMMVDFVRDWPEYKKQLEMLRKKFLNKERIMELLEELREPFWRVTN
ILRDDLREFMTLVPTEKVLVITLEYLANDQELQEFFVYIQSEKFPMVHTI
VEYLKQYKQCLKFIDDRGVNVYTIIKKIHDTLGYPPFEPTNTTRMGVGIH
GLIHDVVAVLPFYDLMALFEKKMETSKDFKTLVTVIRSPVFMNMVHAVQA
WPEYHKLLEILQQKVVDVHRIIELLREISWRGTHSLQEDLNDFMVIFPTK
KVLEIALDYLSNDQELKEFIVYIQSEEFPKIHRVVEYLKEYKNCFKFIND
QGIDVYAIINVIYDILERHPFQPTNTTRMGVGIYGLMDDVVAVLPVNELK
TLFDKKMLTSKGFKSLVTAFHSPEFANTVDSVRAWPEYKKLLESLQRKGV
DVDRIIEHLGALLGLSWRGTPRLQDDLKEFMALVPTDKVLEIALDYLAND
QEVHEFVVYIQSEEFPKIHTIVENLKEYKDCFKFINDQGVDIYAFINEIY
DTMEKHPFHPTNTTRMGVGIYGLIDDVHAVLP

Cubitermes sp. worker+nymph GHZH01097255.1
GLPPFQPRHSTRRGVGINGLIEDLLAVLPLEELKALFDYKLANSEDFKTL
VEGIRSDTLAKIVETLRAIPEYQDLLQKLRDKGVDVDRIIEFVRALFGLS
RKGTRTLQDDLNEFLALIPVDKVLEITLDYLANDVEVQEAVIYLQSEEFY
KILSTVEALPEFKEFVQFINDLGVDVYAII

Cubitermes sp. worker+nymph GHZH01097272.1
LWEISLRETHTLQDDLHEFMALFPTNKVFDIVLEYIKNDKEMLEFFVYIQ
SEEFPPIHRFVEYLKEYKDCFKFINDQGVDVYAFILEIHDILGLPPYQPT
NTPRMGVGIRGLVEDVIAVLPLHEFKALLDKKMETREYLKVVVTAIRIHV
PVLKKMVETVRTWPEYKKLLEILREKRVDADHIIEFLRTLYELSWRGTRR
LQDVLREFMAPVPMDKFFEIVLDYLANDEEVREFVAYMQSEKFPKIHTVV
ENLKEYKDCFKFINDQGVDVYAIINEIHDNLEMHPFQPTNTTRHGVGIYG
LINDVVAVLPFEDWSALLDKKMETKDYFKTLVTTLMSPVFMNMMNTVQAW
PEYNEMVEILRVMFVDVQRIIHLLRELSMRETRSLQDDLKEFMVLFPTDK
VIQITLDYLANDPELHEFFAYIQSEEFPKIHRVVEYLKQYKDCFKFISDQ
GVDVYAFILEIHDILGLPPFQPTNTTRMGFGFYGFIDDVYTVLPLDDLKA
LFDMKMETRQYFKTIVTTIKTIVTTMRSPLFMNMANTVRSWLEYQKLPET
LQQMGVDMEGIIEHLREQFGPDPLYNRR

Indotermes sp. 1 worker GHZW01022852.1
ALDYLANAVEVQEAVVYLQSAEFHLIVLTIEGLQETKNFLNFLVDLDVPA
YEYLNTLHEIIGLPPIVPKNRTRRGVGISGLLDDVLALLPLDDLKALFDE
KLQTSPDFQAFVAGIQSPEFEVILDTLRALPEYQGLLESLREKGVDVDAI
LEFLKTLFGLSRRGTRTLQDDLNDFLALVPTDEILAVALDYLANDVEVQE
AVIYLQSQEFHKIVLTIEGLQETKNFLQFISNTGVDVYGYLNTLHEIIGL
PPYVPKYSVRRGVGINGLLQDIIAVLPVDDLKALF

Indotermes sp. 1 worker GHZW01126462.1
MMKLSIIFVAVLGLACGKPLPSGGVQDDLNDFLEILPTDKLLEIGLDYLY

YDKEVKEFVVNIQSEKFPEINTFVEYLEEYKDCSKLINEYGVDVYEVIKG
IHDFLESHPHEPKNTASMGVGIYGLISNVISVLPVDELKAFYYEKMETRE
YFKTLVKVVKSPVVMKMVDTMRDWPEYQEMLKIYQEKVVDVDRTIELLSK
LYWRGPCKLLEDLKELMTYIPQHEAFEIVLDYLSTDKEVKEFVVYVQSEE
FPKIHKIVEYLKENIYCSQFISEYGLDIYAIISEIHDILGLPPVKPTSTT
HRGAGINGLIDDVIAILPLYDLKSFFEKKMETRESFKTLVTVIQSPEFLD
MMDTVQAWPKYQELLEILRGKGVDVDRYIELLRTLFSLSWRGNYSLRYEL
IRLVTLFPTDKILDIVMDYLYNDNEVKEFIVYIQSEEFPRIHKIVEYLKQ
YKDCSKFINEYGVDIYAVVKQIHDTLRFPPYEPMNTTRMGVGF

Promirotermes sp. worker+soldier GIAF01064537.1
RGVGFNGLLDDIYAILPVEALKALYEEKLQTSPEFQALVGGLLSPEFAGL
VDNLRAKQEYQVLLQSLLDKGIDVVRVVELIKALFGLSKKGTRTLEDDLN
DFVALLPTDQILEIALDYLANDLEVQEAVIYIQSEEFHPILTTVEALQEF
KDLLKYISDLGVDVYTIINQLHEILGLPPFQPRHSTRRGVGFNGLLDDIY
AILPVEALKALYEEKLQTSPEFQALVGGLLSPEFAVSIHCVYLSYVSCCL
WYVHASVCIPDMNALLHPTGTSG

Nasutitermes sp. soldier GIAB01112380.1
IQSPEFAKILDTLRAIPEYQDLLQKLRDKGVDVDRIIELLKALFGLSRKG
TRTLQDDLNDFLALVPVDKVLEIALDYLANDQEVQEAVIYLQSPEFQKIL
TTVEDLKEFKDFLKFLSDLGIDIYTIINTIHDVLGSPPFQPKHSVRRGVG
INGLIDDVINVLPLNELKALFEEKLQTSPDFQALVKAIQSPEFANIVDTL
RALPEYQALLQSLRDKGVDVDRIIELLRALFGLP

Nasutitermes sp. soldier GIAB01104082.1
VDVYAIISEIHYILEGHPFQPTNTTRTGVGIYGLIDDVIAVLPLSEWKAL
IDKKMEASQELKTLVKLIDIPALMSVVDTVRSWPEYYELLEIVQEIVREK
DVDVDRIIELFKQLYRRGTPWLQDSLKEFMALIPTDKVLEISLDYLAYDQ
EVREFIVYIQSEEFPQIHKIVEYLQKYKDCFKYISEYGIDVYEIISEIHY
MLEKHPFQPTNTTRMGVGIYGLISDVIAVIPLDDLAAFLDKKMETGEFFK
NLLTAIRSIDVMKMVDTVRAWPEYQEMLEILREMVPYVERIYKILRELSL
RGIHRVQDDLKEFMALVPTEKVLEITLDYLHEAFVYIQSEEFPKIHKIVE
YLKKYKDCFPIISDQGVDIYGIVNEIRDILGLPPIPPTNVTRMGLIDDVI
AVLPLDDLKVLLDMKMETRVYIKTVVTAIRSPVLVNMVETVQDMVETVQA
LPQYQRLLEILREKGVDVESIIEHLRVIFYPPPP

Amitermes sp. worker GHXC01038018.1
DIIAVLPVDELKALFNEKLETSPDFQALVAGIQSPEFAIIVEKLRALPEY
QDLLQKLRDKGIDVDYFIELLRALFGLSRRGTRTLQDDLNDFLALVPLDK
VLEIALDYLANDPEVQEAVIYLQSEEFHKIITTVEDLKEFRDFVKFINDL
GVDVYAIINTIHDILGLPPFQPRHSTRRGSGLNGLIDDIIAVLPVDELKA
LFNEKLETSPDFQALVAGIQSPEFAIIVEKLRA

Amitermes sp. worker GHXC01011135.1
GVDVYAIINEIYDILERHPFQPTNTTRKGVGIYGLIDDEIAVLPVNLTAL
LGKKMETREIFKTLVTATHSPVFLKMVHTVRAWPEYNEVLEILQVMVVDV
QHIIELFWELYLEGTHSIRDDVKEFMALVPTNKIFEITMDYLAHDPELHE
IFAYIQSEQFPKIHKIVEYLKQYKDCFSFINDEGVDVYAFILEIHDILGL
PPFEPTNTTRMGVGIRGLINDVVAVLPIDDWAALLDKKLETEEFFQNLVT
ANLSSAFVNMVNTVQAWPEYHEVHEILREMVVNVKQITKILWELSLRETR
SLQDDLHDFMALVPTNKVFEVFNIALGYLSNNKELQEFVVYIQSEEFPKI

HKIVEHLKEYKDCFKFINDQGVDMYEIINEIRDILDLPPFQPTNTTRGIQ
GLIDDVVAVLPLDELKALYDKKMETRKYFKALNTLVTALKSPVFVNMVNT
V

Pericapritermes sp. worker GIAE01163624.1
GLNGLIDDVVAVLPVDELKALFDEKLVTSPDFQALVAGIQSDEFGKIVDT
LRALPQYQELLQRLREKGVDVDKFIELLRALFGLSRKGTRTLQDDLNDFL
ALVPTDKILEIALDYLANDPEVQDAVIYLQSEEFHSILLTVEALPEFKNF
VKFISDLGVDIYTIINTIHDILGLPPFQPKHSTRRGFGLNGLIDDVVAVL
PVDELKALFDEKLVTSPDFQALVAGIQSDEFGKIVDTLRALPQYQELLQR
LREKGVDVDKFIELLRALFGLSR

Pericapritermes sp. worker GIAE01058492.1
WEFHRLQDDLKEFMELNPTNKVLEIALDYLAYDEEVRKFVVYIQSEEFPKI
HTIVEHLTEYKDCLKFINDQGVDVYAIVNEIHDKLERHPYQPTNTTHMGV
GFYGLVDDVFAVLPLDDWAALLDKKMETGEFFKTIVTTIRSPVLVKMVDT
VRAMPEYHELLEILQEKVVYGERIIKLVWEISRRGTHTLRNDLKEFMALF
PTDKVIQITLDYLSNDPELQEKFAYIHSEEFPRLHAIVEHFKEYAYCLKF
INDQGVDVYAFVNEIYDIFGLSPFQPTNTTRTGGGIYGLINDVFGVLPLD
DFKTLFYTKMETREYFKTIITTIPFPVFVKTVQTVRAWPEYHELIEIVRE
KDFDVEHIFELFRKLYDREFHRLQDDLKEFMELYPTDNVLEIALDY

Hospitalitermes sp. worker GHZV01006778.1
AVIYLQSPEFEKIITTVEDLKEFKDFLKFLNDLGIDVYSILDTVHDVLGL
PPFKPKHSVRRGVGLNGLIDDVINVLPLDELKALFEQKLQTSPDFKALVA
AIQSPEFANIVNTLRALPEYQDLLQALRDKGVDVDRIIELLRALFGLSRK
GTRTLQDDLNDFLALVPLDKVLEIALDYLANDQEVQDAVIYLQSPEFQKI
LTTVEDLKEFKDFLKFVDGLGVDIYSILNTIHDVIGLPPFKPKHSVRRGV
GLNGLIDDVINVLPLDELKALFEQKLQT

Hospitalitermes sp. worker GHZV01006788.1
MKLPIVFLAVLGLVYGKSLPTRTLQDDLNDFLALVPTNKVLEIYLEYLTN
DPEVQEAIAYLQSEEFHVLLTTVENLKEFKDYVKFMNDLGVDVYELDNQI
HDILGLPTLQPKPSTRRGVGLNGFIDDVINVLPIDKFKALFDEKLQTSPE
FQALVAAIQSPEFVNILDTLRATPEYQDLLQRLQEKGFDVDHFNELFRAL
FGLSQKGTPTLQDDLNDFLALVPMDKVLKIVLDYLSNDKEVREFVVHIQS
EEFPKIHTTIEHLKKYKDCSKFNNDHAVDVYVFINVIHDFLGLPPFQPTN
ATRMGVGIYGLIEDVIAVLPLHDVKALFEV

Promirotermes sp. worker+soldier GIAF01095832.1
FKAYFNKKTETREYFKALLTVIRSPEFVNIMDNVQNLVNTVQNMVYTVQT
WPEYKMLLEIVQEKRVDEDRIVELLKTLYDRSWQGTQRLVDELKQIMAYF
PTDKVLETTLDYLANDEELREFLVHIQSEEFPQIHKIVEHLKEYKNCFKF
INNEGVDVYAIVNEIYEILERHPYQPTNTPRMGVGINGLIHDVVAVIPVE
DLTNHLDKEMQIREFFKPLVTALKSPVLKNMVNTVQAWPEYNELREILRQ
MVSDAEPHIKNLWELSLRGTHNLRQDVKEFMALVPTEQLIQITLDYLTND
PQLQEIYAYIQPEQFPKIHEIVEYVKEYKYCFKFINDEGVDVYEFINEIY
HILNLPPFQPTNTTRMGVGIYGLIDDVVAVLPFSDLMALFDMKMETSKDF
KTLVTIIHSPVFMNMVKTVRAWPEYHQLLGILQEKFVDVEHIIELLREIS
WRGTHSLQDDLKDFMAIFPTDKVLEIALDYLANDQEVKDFVVYIQSEEFH
KIHTVVEYLKEYKDCFKFINDHGVDVYAIINEIHDSLE
